# Children with developmental coordination disorder have less variable motor unit firing rate characteristics across contractions compared to typically developing children

**DOI:** 10.3389/fnhum.2023.1294931

**Published:** 2023-12-07

**Authors:** Maaike Esselaar, Johnny V. V. Parr, Greg Wood, Emma Hodson-Tole

**Affiliations:** ^1^Department of Sport and Exercise Sciences, Manchester Metropolitan University, Manchester, United Kingdom; ^2^Institute of Sport, Manchester Metropolitan University, Manchester, United Kingdom; ^3^Department of Life Sciences, Manchester Metropolitan University, Manchester, United Kingdom,

**Keywords:** Dyspraxia, muscle activity, electromyography (EMG), isometric contractions, HD-EMG, neuromotor control, developmental disorders

## Abstract

**Introduction:**

Understanding the nuances of neuromuscular control is crucial in unravelling the complexities of developmental coordination disorder (DCD), which has been associated with differences in skeletal muscle activity, implying that children with DCD employ distinct strategies for muscle control. However, force generation and control are dependent on both recruitment of motor units and their firing rates and these fine details of motor function have yet to be studied in DCD. The purpose of this study was therefore to compare motor unit characteristics in a small muscle of the hand during low level, handgrip contractions in typically developing (TD) children and children with DCD.

**Methods:**

Eighteen children (9 TD vs. 9 DCD) completed a series of manual handgrip contractions at 10 ± 5% of their maximum voluntary contraction. High density surface electromyography was used to record excitation of the first dorsal interosseus muscle. Recorded signals were subsequently decomposed into individual motor unit action potential trains. Motor unit characteristics (firing rate, inter-pulse interval, and action potential amplitude) were analysed for contractions that had a coefficient variation of <10%.

**Results and Discussion:**

This study found few differences in average motor unit characteristics (number of motor units: TD 20.24 ± 9.73, DCD 27.32 ± 14.00; firing rate: TD 7.74 ± 2.16 p.p.s., DCD 7.86 ± 2.39 p.p.s.; inter-pulse interval: TD 199.72 ± 84.24 ms, DCD 207.12 ± 103 ms) when force steadiness was controlled for, despite the DCD group being significantly older (10.89 ± 0.78 years) than the TD group (9.44 ± 1.67 years). However, differences were found in the variability of motor unit firing statistics, with the children with DCD surprisingly showing less variability across contractions (standard deviation of coefficient of variation of inter-pulse interval: TD 0.38 ± 0.12, DCD 0.28 ± 0.11). This may suggest a more fixed strategy to stabilise force between contractions used by children with DCD. However, as variability of motor unit firing has not been considered in previous studies of children further work is required to better understand the role of variability in motor unit firing during manual grasping tasks, in all children.

## 1 Introduction

Developmental coordination disorder (DCD) affects 5–6% of children (American Psychiatric Association, 2013) and is associated with poor learning and performance of motor skills (Parr et al., 2020a) and appropriate force control. While the cause of DCD remains largely unknown, there is growing evidence that individuals with DCD display fundamental differences in their brain structure (Gill et al., 2022), brain activation patterns (Scott et al., 2021), and possibly how their brain communicates with the contracting muscles controlling force production (as observed in a single participant case study; Parr et al., 2022).

Understanding the nuances of neuromuscular control is crucial in unravelling the complexities of DCD. This disorder has been associated with fundamental differences in skeletal muscle activity, implying that children with DCD employ distinct strategies for muscle control (Fong et al., 2018). To investigate these strategies, researchers have used surface electromyogram (sEMG) recordings, which capture the interference pattern of detected motor unit action potentials. Analysing sEMG signals from children with DCD has revealed they tend to activate their muscles later, for longer durations and with more co-contraction across agonist/antagonist pairs in perturbation based postural balance control tasks (Williams and Castro, 1997; Raynor, 2001; Johnston et al., 2002; Williams, 2002; Geuze, 2003; Fong et al., 2013) and play-based activities like throwing and catching a ball (Fong et al., 2015) and uni- and bi-lateral aiming tasks (Huh et al., 1998).

Although these studies provide insight into the general patterns of muscle activity that may impact task performance, the measures used from the sEMG do not reveal details of the neuromuscular control used to produce the resulting joint force profiles (Martinez-Valdes et al., 2018). This will depend on the behaviours of the individual functional units within the active muscles. In skeletal muscle, these functional units are termed motor units and comprise the α-motoneuron, its axon and the group of innervated muscle fibres (Sherrington, 1925). An increase in force output from a muscle can be achieved by either recruiting more motor units or by increasing the firing rate of the already recruited motor units. sEMG amplitude measures do not reveal the number of recruited motor units nor their firing rates because the signal that is measured is the interference pattern of all the detected motor unit action potential shapes and firing rates (Martinez-Valdes et al., 2018). Traditionally, studying individual motor unit behaviours was only possible using invasive, intramuscular needle or fine-wire electrode techniques (Merletti and Farina, 2009). This has made it unfeasible to study motor unit behaviours in some populations, including children.

Advances in the availability of algorithms that can decompose sEMG signals into the individual motor unit action potential trains, do now, however, make it possible to extract such information from this signal, which can be recorded less invasively (De Luca et al., 2006; Holobar and Zazula, 2006; Pope et al., 2016). The currently available algorithms rely on availability of multiple (i.e., more than two) signals, recorded simultaneously from surface electrodes placed relatively close to each other on the same muscle (typically known as high density EMG, HD-EMG). The proximity of the recording sites provides multiple views of the same motor unit action potentials and, in various ways, the algorithms use the similarity and differences in signal information content to estimate the action potential shapes and firing instances of detected units that sum to produce the muscle behaviour. Decomposing HD-EMG signals, therefore provides a means of revealing some of the individual motor unit behaviours that contribute to a given motor task using a signal that can be easily recorded in children (and other previously inaccessible patient populations).

Decomposing EMG signals into constituent motor unit action potential trains has enabled differences in motor unit behaviours to be identified in adult patient groups where motor control is affected by pathology. For example, in stroke survivors, paretic muscles exhibit lower motor unit firing rates than non-paretic (Rosenfalck and Andreassen, 1980; Young and Mayer, 1982; Mottram et al., 2014) even for the same level of force production (Gemperline et al., 1995). This suggests that paretic muscles need to recruit more motor units to produce a given force, likely influencing the metabolic cost and muscle fatiguability. In people living with Parkinson’s disease motor unit firing rate has been found to be the same as healthy controls, however significant differences in the variability of firing rate are found (Wilson et al., 2020). This suggests that in mild-moderate Parkinson’s disease motor dysfunction is linked to variability in motor output. These two examples highlight how different conditions associated with movement impairment, are underpinned by differences in the motor system responses to a given task. To date however, there have been few studies of motor unit behaviours in children (Herda et al., 2019; Miller et al., 2019), and we could not find any that had compared motor unit behaviours in typically developing (TD) children and those with DCD. Given the fundamental connection between motor unit behaviour and the ability to meet the time varying force requirements of any movement task, this seems a significant gap in the current literature.

The purpose of this study was therefore to investigate motor unit characteristics in a small muscle of the hand during low level (10% maximum voluntary contraction) isometric handgrip contractions in TD children and children with DCD. Handgrip was selected as the study task due to its direct relevance to daily functional tasks (e.g., squeezing a toothpaste tube, opening food jars). The first dorsal interosseus (FDI) was selected as the muscle to be studied as it is easily palpated and offers an accessible location for required HD-EMG sensors to be secured, even in smaller children. In addition, there is a strong correlation between grip strength and finger strength (*r* ≥ 0.93), with the fingers on the radial side of the hand contributing ∼60% to overall grip strength (MacDermid et al., 2004). The index finger accounts for 25% of total grip strength (MacDermid et al., 2004) and hence FDI can be considered to contribute to the task studied. Assessing the firing rate and relative range of action potential sizes from recorded HD-EMG signals will provide insight into the neuromuscular control strategy associated with handgrip and might highlight factors contributing to control deficits in children with DCD.

## 2 Materials and methods

### 2.1 Participants

Thirty-eight participants, aged 7–12 years, were recruited for the study which had been approved by the local ethics committee in the Faculty of Science and Engineering, Manchester Metropolitan University (ethics number 41284). Children in the DCD group were recruited via social media, local support groups and via the Dyspraxia Foundation. The TD group was recruited via a local scout group, siblings of the children with DCD and from the family of student and staff members of the Manchester Metropolitan University.

Children in the DCD group were classified based on the Diagnostic and Statistical Manual of Mental Disorders (DSM-5) criteria (American Psychiatric Association, 2013), whereby they exhibit substandard motor ability, relative to their chronological age. Prior to data collection, parents completed the Developmental Coordination Disorder Questionnaire (DCDQ, Wilson et al., 2009) to confirm that their child had significant movement difficulties that interfered with their child’s daily lives. Co-occurrence with ADHD was also recorded using the Vanderbilt ADHD Diagnostic Parent Rating Scale (Wolraich et al., 2003). Finally, parents confirmed that their child did not suffer from any general medical condition known to affect sensorimotor function and had no diagnosed learning difficulties.

For the DCD group, children who scored 57 or below on the DCDQ (classified as suspected DCD) and below 18 on the ADHD rating scale (indication no ADHD) were invited to take part in the study. For the control group a score of 58 or higher on the DCDQ (indication no DCD) and below 18 on the ADHD (indication no ADHD) were invite to the lab. The DCDQ score was therefore used as an initial indication of DCD, which was subsequently confirmed with the Movement Assessment Battery for Children–2 (MABC-2) (see section “2.2.1 Assessment of motor impairment”).

On visiting the laboratory, participants and guardians were shown a pictorial overview of the study and were given the chance to ask questions and discuss what would be required of them. After, participants provided written assent and the guardian completed a written consent form.

### 2.2 Data collection procedures

#### 2.2.1 Assessment of motor impairment

The MABC-2 is a test of motor impairment. The test assesses three domains: Manual Dexterity, Balance and Aiming and Catching with eight tasks in total. A total MABC-2 test score of up to 56 reflects a percentile score of 5% or less and denotes a significant movement difficulty, a total test score between 57 and 67 with a percentile score between the 5th and 15th suggest that the child is “at risk” of having a movement difficulty, any score above 67 or above the 15th percentile denotes no movement difficulty. In this study, children who scored at or below the 5th percentile for the total MABC-2 score were included in the DCD group while those who scored at or above the 20th percentile were allocated to the TD group.

#### 2.2.2 Assessment of force production and motor unit activity

Participants sat at a table with 268.1 mm × 476.6 mm size screen (Iiyama Co., Ltd, Iiyama, Japan) located 60 cm in front of them. The dominant hand was assumed to be the hand the child used to sign the assent form, and this was the hand with which all testing was completed. To collect surface EMG data, a four-pin surface array sensor (Delsys, Inc., Natick, MA, USA) was attached to the mid-belly region of the FDI muscle of the dominant hand. The diameter of each pin is 0.5 mm and they are placed at the corners of a 5 mm × 5 mm square. Before the sensor was placed, the surface of the skin was prepared by shaving, applying, and removing tape to remove dead skin and dampening the skin with a paper towel. Data was recorded at 20 kHz using the EMGworks Acquisition (v. 4.8.0, Delsys, Inc., Natick, MA, USA).

**FIGURE 1:**
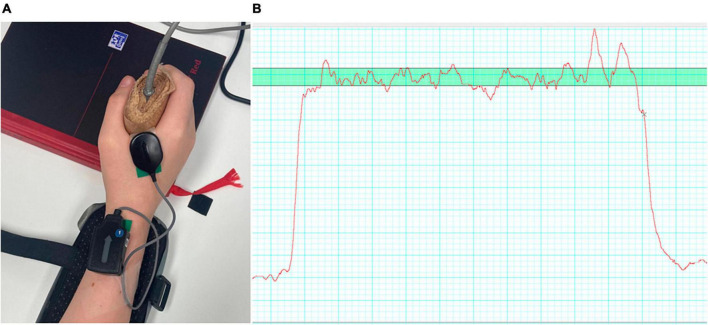
**(A)** Showing the EMG electrode placement on the hand of a participant; **(B)** and an example of a force trace (red line) with the target zone (green, horizontal bar) also shown.

The experimental protocol was based on a handgrip task, whereby participants were asked to repeatedly squeeze a hand-dynamometer (Parr et al., 2022, 2023). The dynamometer was attached to a PowerLab 4/25 T (AD Instruments, Bella Vista, NSW, Australia) that recorded the hand contraction force (in kilogrammes) via Labchart 8 software (ADinstruments, Sydney, NSW, Australia) at a sampling rate of 1000 Hz. Participants first completed three maximum voluntary contractions (MVC) with 1-min break in between each attempt. The force profile was presented to them on the screen using the Labchart interface. The peak force value achieved across the three recorded MVCs was used to set the target zone, 10% MVC ± 5%, which was displayed as a 15 mm thick green band that extended the entire width of the screen (Figure 1).

During each trial participants were asked to squeeze the dynamometer, so that the force trace remained, as steady as possible and within the target zone. A single trial comprised 6 × 10-s-long contractions each separated by 10 s of rest. Feedback about the steadiness and accuracy of the force was provided during the practice trials and between trials. An audible tone, controlled through a custom PsychoPy (Peirce et al., 2019) script, signalled the start/end of each contraction. Eleven trials were completed in total (including one practice trial at the start) with 1 min rest between the trials for a total of 66 contractions per participant.

### 2.3 Data analysis

#### 2.3.1 Force data

Force data were extracted from the Labchart software, and each contraction was analysed for accuracy and steadiness of force production. Force accuracy was defined as the percentage of time participants were able to maintain their force output within the target zone. Force steadiness was defined as the coefficient of variance (CoV), calculated as the percentage of the force standard deviation to the mean force value for that contraction. Both force accuracy and steadiness were assessed from 1 to 10 s following the auditory “go” stimulus, as the first second was likely to contain dynamic fluctuations in force as participants ramped-up their force output. For each participant, we also calculated the standard deviation of force accuracy across all contractions (Accuracy SD), to express their contraction-to-contraction variability in task accuracy. These data were analysed using a bespoke Matlab (version R2021a) script.

Across recorded trials not every contraction attempt produced steady force outputs, with some showing consistently large fluctuations in magnitude. Therefore, to identify whether motor unit behaviours differed between the two groups it was important to ensure analysed data represented comparable force production behaviour. Thus, only contractions where the force CoV was 10% or below were included in the analysis. This threshold was defined prior to data analysis and based on previous work reported in Smits-Engelsman et al. (2003). The data of children who had at least 20 trials with CoV < 10% were included in the study.

#### 2.3.2 Motor unit characterisation

Action potentials were extracted from recorded sEMG signals for each contraction from 1 to 10 s following the auditory “go” stimulus (as per force data) to provide individual motor unit action potential firing trains. This was achieved using the precision decomposition (PD) III algorithm described by De Luca et al. (2006) and commercially available as NeuroMap software (v. 1.2.2, Delsys, Inc., Natick, MA, USA). The analysis processes involved pre-processing which includes filtering data at 20 Hz and a baseline correction, feature extracting, template matching, decomposing the EMG signal by matching identified MU templates onto the data and extracting them. The Neuromap Explorer software was then used to select, and export for further analysis, the motor unit information pertaining to units decomposed with ≥85% accuracy within valid contractions (where force CoV < 10%). The accuracy measure provided by the software is based on the decompose-synthesise-decompose-compare approach described by De Luca and Contessa (2012).

From exported motor unit data, the following outcome measures were recorded for each participant: average number of motor units identified per contraction; their average firing rate (FR) (expressed as pulses per second; p.p.s.) and average inter-pulse-interval (IPI) (in milliseconds). As both average FR and average IPI are derived from the series of instantaneous measures generated by decomposition it is not possible to directly convert between the two, because such conversion is only possible between single data points (Johnson, 1994). The variability of IPI within each individual contraction was reported as the CoV of IPI (IPI-CoV, reported as the ratio of standard deviation: mean). By contrast, the standard deviation (SD) was calculated to determine the contraction-to-contraction variability of IPI (IPI-SD), IPI CoV (IPI-CoV-SD), and firing rate (FR-SD) across all contractions.

In addition, to investigate the diversity of characteristics in the recruited motor unit pool within participants, the average motor unit action potential amplitude (mV), average FR and average IPI across contractions were quantified for units within the 10th and 90th percentile for motor unit action potential amplitude. To account for differences in motor unit action potential amplitude stemming from other, extraneous, factors (e.g., different subcutaneous adipose thickness) the ratio of the average smallest and largest action potential amplitudes (10th vs. 90th percentile) was calculated in each participant, essentially normalising data to facilitate comparison across groups.

#### 2.3.3 Statistical analysis

For the within participant measures described above, a Shapiro-Wilk test confirmed the distribution of all dependent variables were normal (*W* = 0.898–0.980, *p* = 0.053–0.953). Independent *t*-tests were chosen to assess between group differences in age, MVC, force control (Force CoV in%, Accuracy, Accuracy SD), and measures describing motor unit characteristics (FR, IPI, IPI-CoV as ratio, FR-SD, IPI-SD, IPI-CoV-SD). Paired *t*-test was used to test for differences between firing of the biggest and smallest motor units within the two groups. Unless otherwise mentioned all tests were two tailed.

For all statistical tests, significant differences were considered to occur when *p* < 0.05. No adjustments for multiple comparisons were made, on the basis that this is an initial exploratory investigation of this topic and increasing the chance of a type II error could risk missing potentially useful findings (Rothman, 1990). The lack of previous motor unit data from TD children and those with DCD means it was not possible to complete *a priori* power calculations, and sample size was selected based on previous motor unit assessments in TD children (Miller et al., 2018, 2019). Therefore, here the effect size associated with each comparison (described above) was also calculated as Cohen’s *d*, with ≤0.1 considered small, 0.2–0.3 medium, 0.4–0.5 large effect size (Cohen, 1988). Average and standard deviation values for TD and DCD groups are reported, alongside the confidence interval (CI), in the following results.

## 3 Results

### 3.1 Participant characteristics

Of the 38 participants recruited 18 had a minimum 20 trials with CoV < 10% of these nine satisfied the criteria for DCD (MABC-2 score at or below the 5th percentile) (Table 1). The average age of the DCD group was 10.9 years old (range 10 to 12 years) and 9.4 years (range 7–12 years) for the TD group (Table 1). The TD group was significantly younger than the DCD group (*t* = −2.35, *p* = 0.032, 95% CI = −2.75 to −0.144, *d* = 1.27).

**TABLE 1:** Mean movement ABC-2, DCDQ and ADHD rating scale scores and age within participant group.

	Group	
Measure	DCD (*N* = 9)	TD (*N* = 9)
MABC-2 Overall percentile score (percentile)	1.78 (1.39)	98.11 (1.27)
MABC-2 aiming and catching (percentile)	6.56 (5.98)	91.56 (4.90)
MABC-2 balance (percentile)	4.72 (7.79)	90.89 (6.48)
MABC-2 manual dexterity (percentile)	7.72 (6.45)	93.11 (3.33)
DCDQ score	24.8 (11.9)	65 (7.2)
ADHD diagnostic parent rating scale score	17.4 (1.4)	15.3 (1.8)
Age (Years)	10.89 (0.78)	9.44 (1.67)
Sex split	7 male	6 male

Standard deviation values are shown in parentheses. NM indicates not measured.

### 3.2 Force control characteristics

**FIGURE 2:**
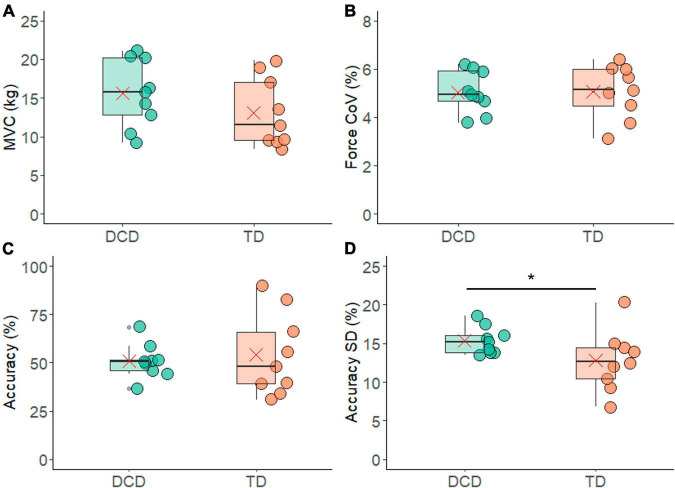
Box and whisker plots illustrating force measurements from each group. **(A)** Maximum voluntary contraction. **(B)** Co-efficient of variation (CoV) of force. **(C)** Force accuracy. **(D)** Standard deviation of force accuracy. In each figure mean and median group values are denoted by the × symbol and horizontal line, respectively. The edges of the boxes represent the 1st and 3rd quartile values. Grey circle symbols indicate outlier values (identified in gg-plot as any values over 1.5 times the interquartile range over the 75th percentile or any values under 1.5 times the interquartile range under the 25th percentile), with the whisker edges representing the minimum and maximum values (excluding outliers). Significant differences (*p* < 0.05) between groups are denoted by the horizontal bar and asterisk (*).

The TD group had an average MVC of 13.06 ± 4.46 kg and the DCD group had an average MVC of 15.35 ± 4.38 kg (Figure 2A). These values were not significantly different between the two groups (*t* = −1.213, *p* = 0.243, CI = −6.95 to 1.89, *d* = 0.6).

In total 453 contractions from the TD group and 500 contractions from the DCD group (both out of a total of 9 participants × 6 contractions × 11 trials = 594 contractions) met the criteria of a CoV in force of less than 10% and were included in the analysis. The TD group had an average of 50.33 ± 11.92 valid contractions per person, while in the DCD group an average of 49.22 ± 13.30 valid contractions per person. The number of contractions included did not differ significantly between the two groups (*t* = 0.19, *p* = 0.85, 95% CI = −11.51 to 13.73, *d* = 0.1). In addition, the CoV for force was 5.06 ± 1.11% for the TD group and 5.04 ± 0.88% for the DCD group. These values were not significantly different between the groups (*t* = 0.048, *p* = 0.962, 95% CI = −0.98 to 1.02, *d* = 0.9) (Figure 2B).

When examining force accuracy, the TD group were accurate for 54 ± 21% of the contraction time while the DCD group were accurate for 50 ± 8% of the contraction time (Figure 2C). There was no statistically significant difference found between the two groups (*t* = 0.43, *p* = 0.67, CI = −0.13 to 0.20). However, it is noteworthy that the standard deviation of the force accuracy was significantly greater in one-sided testing (*t* = −1.84, *p* = 0.042, 95% CI = −0.05 to 0.003, *d* = 0.9), with the DCD group having a mean standard deviation of 15.3 ± 1% which was greater than that of the TD group, 12 ± 3% trial time (Figure 2D).

### 3.3 Motor unit characteristics and behaviours

**FIGURE 3:**
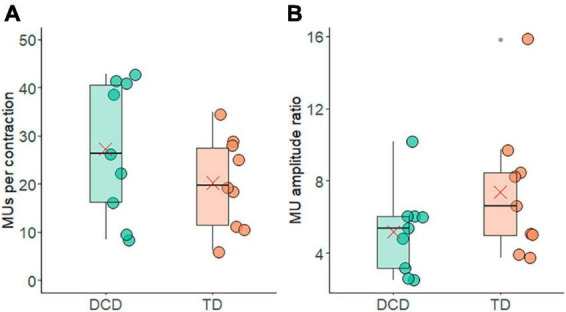
Box and whisker plots illustrating average motor unit characteristics from each group. **(A)** Number of motor units per contraction. **(B)** Ratio of the motor unit action potential amplitude from the largest and smallest motor units. In each figure mean and median group values are denoted by the × symbol and horizontal line, respectively. The edges of the boxes represent the 1st and 3rd quartile values. Grey circle symbols indicate outlier values (identified in gg-plot as any values over 1.5 times the interquartile range over the 75th percentile or any values under 1.5 times the interquartile range under the 25th percentile), with the whisker edges representing the minimum and maximum values (excluding outliers).

The average number of motor units identified per contraction with accuracy ≥85% was 20.24 ± 9.73 in the TD group and 27.32 ± 14.00 in the DCD group (Figure 3A). The average number of motor units per contraction did not differ between the groups (*t* = −1.25, *p* = 0.116, CI = −19.13 to 4.97, *d* = 0.6). The average ratio between the largest-smallest average motor unit action potential amplitude of the TD group was 7.40 ± 1.27 and 5.29 ± 0.98 for the DCD group. This difference was not significant (*t* = 0.19, *p* = 0.85, 95% CI = −11.51 to 13.73, *d* = 0.6) (Figure 3B).

**FIGURE 4:**
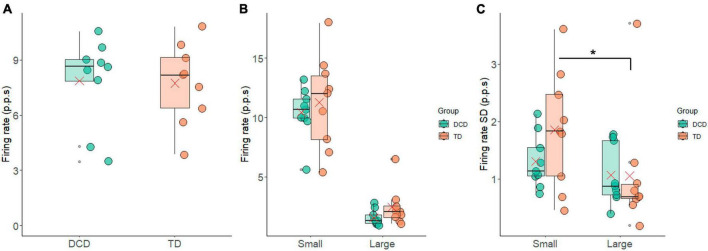
Box and whisker plots illustrating the firing rate measures from each group. **(A)** Average firing rate. **(B)** Average firing rate of the 10% largest and smallest motor units. **(C)** Standard deviation in firing rate of the 10% largest and smallest motor units. In each figure mean and median group values are denoted by the × symbol and horizontal line, respectively. The edges of the boxes represent the 1st and 3rd quartile values. Grey circle symbols indicate outlier values (identified in gg-plot as any values over 1.5 times the interquartile range over the 75th percentile or any values under 1.5 times the interquartile range under the 25th percentile), with the whisker edges representing the minimum and maximum values (excluding outliers). Significant differences (*p* < 0.05) between groups are denoted by the horizontal bar and asterisk (*).

The average FR exhibited notable similarity between the groups, with 7.74 ± 2.16 p.p.s. for the TD group and 7.86 ± 2.39 p.p.s. for the DCD group (Figure 4A). Again, there was no statistically significant difference between these values (*t* = −0.11, *p* = 0.91, 95% CI = −2.40 to 2.16, *d* = 0.1). The FR of the 10% largest and smallest motor units are shown in Figure 4B. In the TD group the mean FR was 2.22 ± 1.75 p.p.s. and 10.82 ± 3.77 p.p.s. for largest and smallest motor units, respectively. In the DCD group, the 10% largest units fired at 1.69 ± 0.68 p.p.s., while the smallest units fired at 10.25 ± 2.34 p.p.s. There was a significant difference in the firing rate of the 10% smallest and largest motor units within the TD group (*t* = 8.07, *p* < 0.001, CI = 6.14 to 11.06, *d* = 2.7) and within the DCD group (*t* = 10.45, *p* < 0.001, CI = 6.55 to 10.56, *d* = 3.9). The between group difference for the FR of the 10% smallest motor units was not significant (*t* = 0.35, *p* = 0.731, CI = −2.93 to 4.07, *d* = 0.2) nor was the FR of the 10% largest motor units (*t* = 0.74, *p* = 0.469, CI = −0.99 to 2.03, *d* = 0.4).

The average FR-SD of the smallest 10% was 1.86 ± 1.02 p.p.s. and 1.25 ± 0.47 p.p.s. for the TD and DCD group, respectively. For the largest 10% the average FR-SD was 1.05 ± 1.03 p.p.s. for the TD group and 1.01 ± 0.54 p.p.s. for the DCD group (Figure 4C). There was a significant difference in the FR-SD between the smallest and largest motor units in the TD group (*t* = 2.61, *p* = 0.031, CI = 0.09 to 1.51, *d* = 0.9), this difference was not significant in the DCD group (*t* = 1.05, *p* = 0.33, CI = −0.33 to 0.82, *d* = 0.4).

**FIGURE 5:**
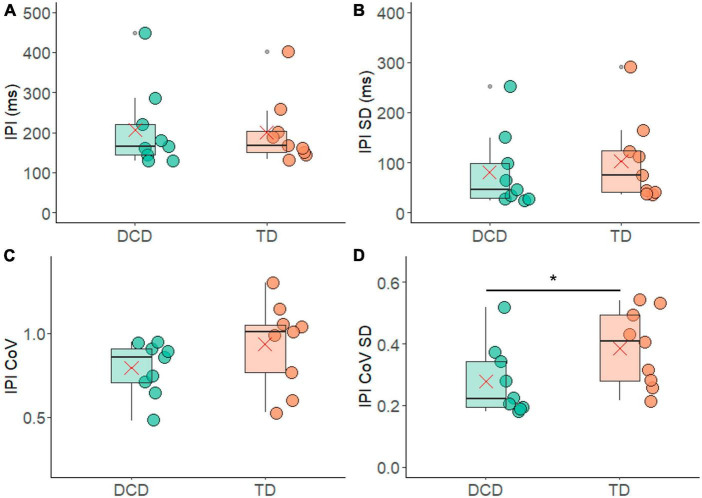
Box and whisker plots illustrating the inter pulse interval measures from each group. **(A)** Average inter-pulse interval (IPI). **(B)** Standard deviation of the average inter-pulse interval. **(C)** Co-efficient of variance of the inter-pulse interval. **(D)** Standard deviation of co-efficient of variance of the inter-pulse interval. In each figure mean and median group values are denoted by the × symbol and horizontal line, respectively. The edges of the boxes represent the 1st and 3rd quartile values. Grey circle symbols indicate outlier values (identified in gg-plot as any values over 1.5 times the interquartile range over the 75th percentile or any values under 1.5 times the interquartile range under the 25th percentile), with the whisker edges representing the minimum and maximum values (excluding outliers). Significant differences (*p* < 0.05) between groups are denoted by the horizontal bar and asterisk (*).

The FR results were also reflected in the average IPI measures, where no meaningful differences emerged between the TD group (199.72 ± 84.24 ms) and the DCD group (207.12 ± 103 ms) (*t* = −0.167, *p* = 0.870, 95% CI = −101.79 to 86.9, *d* = 0.1) (Figure 5A). The average IPI-SD was slightly smaller in the DCD group (179.40 ± 129.40 ms) compared to the TD group (182.31 ± 79.56 ms), a difference that was not statistically significant (*t* = 0.59, *p* = 0.57, 95% CI = −58.21 to 102.66, *d* = 0.3) (Figure 5B). The IPI-CoV was also slightly smaller in the DCD group (0.79 ± 0.16) compared to the TD group (0.94 ± 0.25) (Figure 5C), and while there was a trend of significance during one-sided testing (*t* = 1.443, *p* = 0.080, 95% CI = −0.6 to 0.35, *d* = 0.7) it failed to reach significance. However, the IPI-CoV-SD was significantly lower in the DCD group (0.28 ± 0.11) compared to the TD group (0.38 ± 0.12) (Figure 5D) for one-sided testing (*t* = 1.93, *p* = 0.036, 95% CI = −0.01 to −0.22, *d* = 0.9).

## 4 Discussion

This study was the first to examine the number of detected motor units and their characteristics (ratio of action potential sizes, FR and IPI) in the FDI during low level (10% MVC) handgrip isometric contractions in TD children and children with DCD. This study found few differences in most of the motor unit characteristics when force steadiness was controlled for.

There were no differences in MVC magnitude between the DCD and TD groups. The selection of contractions in which the force produced was maintained within the target window with the CoV < 10% also meant there were no significant differences in the force steadiness profile between groups. This indicates that all children whose data were included in the analysis were able to complete the task, and to do so with similar proficiency, given that the average number of contractions that met the analysis inclusion criteria was ∼50/66 in both groups. However, the percentage of time participants were able to maintain the target force output (force accuracy) was quite low in both groups (Figure 2C), indicating the task did provide a degree of challenge to both groups. Observation of the participants leads us to believe this reflects the challenge of moving the force into the target window at the onset of the contraction, rather than maintaining the force once the target was achieved. It should however be noted that the TD group was significantly younger than the DCD group, which needs to be considered when interpreting findings.

Previous research has shown that as children mature, their skill at controlling force increases. For example, Smits-Engelsman et al. (2003) showed that children, aged 5–12 years, were able to perform an isometric finger press task with minimal accuracy deviation (2.4%) and no association between the accuracy deviation and age. However, they found that the force variability (measured as CoV) decreased significantly with age. Here we found no differences in the force variability (measured as CoV) nor in the accuracy between the DCD group and the TD group (Figure 2) when controlling for force stability as defined by CoV < 10%, despite the DCD group being significantly older than the TD group (∼1.5 years older). The matching of force steadiness and accuracy performance between the DCD and TD groups could suggest a delay in the development of neuromotor strategies to stabilise force production in children with DCD. This is in agreement with earlier work by Smits-Engelsman et al. (2008), who also found that the standard deviation and the CoV in index finger press isometric force contractions were similar between younger TD children (7–9 year-olds) and older children (11 year-olds) with DCD.

Greater variability in force output has functional implications, for example linking to serious handwriting problems (Smits-Engelsman et al., 2001). This variability has been attributed to a high level of noise in the neuromotor system, making it more difficult to complete perception-action calculations and hence successfully complete fine motor tasks (Smits-Engelsman et al., 2008). Contamination of the neuromotor signal can occur from sources both internal and external to the nervous system (van Galen et al., 1993; van Galen and de Jong, 1995). Within the peripheral nervous system, neuromotor noise can be introduced through recruitment and/or rate coding of motor units. Here, we show that (for the FDI muscle during a handgrip task) the number of motor units detected, and their size, firing rate and inter-pulse interval did not differ between the DCD and TD group. As such, recruitment of units and the average firing characteristics (i.e., rate coding) of the recruited motor unit pool do not seem to be the source of any differences in neuromotor noise that might exist between TD children and those with DCD.

However, differences were found in the variability of motor unit firing statistics, i.e., variability in the rate coding. In contrast to what may be predicted based on the neuromotor noise theory, less variability was found in the motor unit firing statistics between contractions. Specifically, the variability in IPI-CoV between contractions, represented by IPI-CoV-SD, was significantly smaller in the DCD group compared to the TD group (Figure 5). In addition, the TD group exhibited a significant difference in the standard deviation of firing rate between the largest and smallest motor units (Figure 4). This difference was not however found in the DCD group, again indicating smaller contraction-contraction variability in this group. The children with DCD therefore repeatedly produced the same force output patterns with less variance in the firing rate statistics in the recruited motor unit pool across contractions. The implication of this smaller variance is unclear, however it may reflect patterns of motor unit behaviour were more fixed (e.g., fewer motor unit combinations detected) across contractions. If so, this could increase fatiguability if the task were to be repeated over very extended periods of time.

It is important to note that the magnitude of differences in variance found here are quite small (although the effect size was large in some cases) and come from a relatively small sample size. However, when taken together with the lack of consideration of variability in previous studies of motor unit firing in children (Miller et al., 2018, 2019; Herda et al., 2019), it is suggested that further studies are required to better understand the role of variability in motor unit firing during manual grasping tasks, in both TD children and those living with DCD.

Indeed, wider study of the role of motor system variability within the context of the greater movement variability that is a hallmark of DCD (Geuze and Kalverboer, 1987; Williams et al., 1992; Volman and Geuze, 1998; Bo et al., 2008; Mackenzie et al., 2008; Smits-Engelsman et al., 2008; Roche et al., 2016; Golenia et al., 2018; Parr et al., 2020a,b) seems important. The influence of motor unit recruitment on the relationship between within and between movement variability should also be considered. This is considering our finding that while the children with DCD had the same accuracy level, there was greater variation in their contraction-to-contraction accuracy (standard deviation of the accuracy, Figure 2). This suggests, that while their motor recruitment strategies were on average as successful as those of the TD children, they may constrain the adaptability that enables fine, contraction-to-contraction adjustments. Such exploration would benefit from consideration of the temporal characteristics of variability which have been applied in the study of gait dynamics (Hausdorff, 2007) and dynamics of muscle activity (Wakeling and Hodson-Tole, 2018; Ferrari et al., 2020), to provide further insight into the moment-to-moment adjustments in motor output that facilitate smooth, coordinated movement patterns.

This study is not without its limitations. This is the first time that motor unit behaviour has been assessed in children with DCD via high density surface EMG. Therefore, it was impossible to calculate an *a priori* power and sample size calculation. The relatively small sample size could therefore have contributed to some of the effect sizes being small, while others are strong to very strong. Because there were no differences in the number of trials that were included and the number of detected motor units between the two groups, it is suggested that the HD-EMG sensor worked equally well in both TD and DCD children. This provides confidence that the similarities and differences found reflect the underlying neurophysiological functioning of studied children, and not factors related to our experimental set up. However, the motor unit characteristics reported here are only from one, small hand muscle. The handgrip forces recorded reflect the sum of several muscles in the hand and forearm. As we record only from the FDI caution should be taken when interpreting these results.

In conclusion, this study found that when controlling for CoV in an isometric handgrip task, children with DCD performed as well as their TD counterparts. The underlying muscular control only differed in the contraction-contraction variance of the motor unit firing statistics. Therefore, the underlying motor unit recruitment patterns of TD children and children with DCD do not seem to differ. In contrast, features of motor unit rate coding across contractions did. This difference may indicate that the children with DCD proficiently achieved the task by employing a different strategy in relation to the neural drive received by the recruited motor unit pool. However further work is required to confirm this finding, and to identify whether it is a general feature of neuromotor behaviour across other muscles of the hand and arm.

## Data availability statement

The original contributions presented in the study are included in the article/Supplementary material, further inquiries can be directed to the corresponding author.

## Ethics statement

The studies involving humans were approved by the local Ethics Committee in the Faculty of Science and Engineering, Manchester Metropolitan University (Ethics number 41284). The studies were conducted in accordance with the local legislation and institutional requirements. Written informed consent for participation in this study was provided by the participants’ legal guardians/next of kin.

## Author contributions

JP: Conceptualization, Funding acquisition, Methodology, Software, Visualization, Writing – review and editing. GW: Conceptualization, Funding acquisition, Project administration, Writing – review and editing. EH-T: Conceptualization, Funding acquisition, Methodology, Writing – original draft, Writing – review and editing. ME: Conceptualization and development, Design of methodology, Review, Revision and approval of manuscript, Data curation, Formal analysis, Writing original draft, Software and Visualisation.
